# Exploring the educational needs of patients with cutaneous lymphoma using an educational needs assessment tool

**DOI:** 10.3389/fonc.2024.1433821

**Published:** 2024-08-07

**Authors:** Lina U. Ivert, Anna H. Winther, Pontus Jonsson, Hanna Brauner

**Affiliations:** ^1^ Dermatology and Venereology Unit, Department of Medicine Solna, Karolinska Institutet, Stockholm, Sweden; ^2^ Department of Dermatology, Karolinska University Hospital, Stockholm, Sweden

**Keywords:** mycosis fungoides (MF), cutaneous T-cell lymphoma (CTCL), educational needs, patient education, self-reported questionnaire

## Abstract

**Background:**

Cutaneous T-cell lymphomas (CTCL) are a group of rare non-Hodgkin lymphomas characterized by initial localization of malignant T-lymphocytes in the skin. Support and information from nurses and patient support groups have proven useful for patients with CTCL, but little is known about the educational needs of these patients.

**Objectives:**

To investigate the self-reported educational needs among CTCL patients using an educational needs assessment tool and to explore differences related to sex, age, disease duration, clinical stage, and education.

**Methods:**

This observational single center study analyzed 70 patients with CTCL in routine dermatological outpatient care. The patients were asked to complete a questionnaire to capture their educational needs in regard to CTCL. The questionnaire was inspired by the educational needs assessment tool, designed and validated for patients with rheumatoid disease. The questionnaire included a general question, “In general, how much information do you want to receive about your lymphoma disease?”, and five domains covering information relating to disease process (6 items), treatment (4 items), feelings (2 items), self-management of itch, sleep, and rest (2 items), and support systems (3 items). The domain scores ranged from 0 to 18 (total score from 0 to 51). Each domain score was presented as a mean percentage of the maximum possible domain score.

**Results:**

When asked “In general, how much information do you need?”, females wanted to know more compared with males (2.6 vs. 2.1, p=0.006), and patients with higher education wanted to know more than patients with lower education (2.5 vs. 2.0, *p=*0.025). The domains concerning treatment (80%) and disease process (75%) revealed the greatest needs for education. Patients with a disease duration <2 years reported a greater educational need for the domain support system, compared with patients with longer disease duration. Patients with lower education reported a greater educational need about feelings compared with patients with higher education.

**Conclusions:**

CTCL patients in the cohort, particularly females, expressed a need for education, especially regarding disease process and treatment. A deeper understanding of the educational needs would enable healthcare providers to give personalized information.

## Introduction

Cutaneous T-cell lymphomas (CTCL) are a group of rare non-Hodgkin lymphomas characterized by initial localization of malignant T-lymphocytes in the skin. Several subtypes exist, with the most common form, mycosis fungoides (MF), accounting for nearly 60% of all CTCL cases (1). CTCL present with skin alterations that can cause alopecia, tumors, pruritus, and pain, which can impact patients’ health-related quality of life (QoL), especially in late-stage disease (2, 3). Beyond physical symptoms, anxiety and depression scores have been reported to be higher in MF patients compared with healthy controls (4). Most patients with MF experience an indolent disease course, but 15% progress to advanced-stage disease within five years of diagnosis (5, 6). The prognosis for the patients with advanced-stage disease is poorer, with 10-year overall survival rates of 15–53% (2). Therapeutic options include both skin-directed and systemic regimens, but the available treatments rarely induce long-term remission (7). Patients with advanced disease may achieve prolonged survival through chemotherapy and autologous haemopoietic stem cell transplant (8). As the majority of CTCL patients have a good prognosis, minimizing symptoms, preventing progression, and maintaining QoL are important treatment goals in CTCL.

MF/Sézary syndrome patients worry that their disease may get worse, and have low confidence regarding disease management (9). This highlights some of the educational needs among patients with CTCL. Emotional support and ongoing education provided by nurses to patients with CTCL can have a significant positive impact on the patients’ physical and emotional needs (10). In addition, patient support groups can provide useful information and peer support. However, a growing number of internet sources may spread health-related misinformation (11, 12). To the best of our knowledge, data on educational needs among patients with CTCL are scarce. Adequate education regarding the disease can help patients in disease management, coping with symptoms, and staying adherent to treatment. The aim of this study was to investigate the educational needs among patients with CTCL, with particular focus on MF, and to explore differences related to sex, age, disease duration, clinical stage, and education.

## Patients and methods

### Study design

This observational cross-sectional study was based on data from the local cutaneous lymphoma register at the dermatology clinic of Karolinska University Hospital, Stockholm, Sweden. Data from 15 October 2019 through 30 May 2023 were analyzed. Patients were included in the register during regular clinical visits to the dermatology clinic and data were analyzed at inclusion, which could be at the first visit or after several years of ongoing treatment.

### Study population

The inclusion criteria were: (i) age =18 years, with ability to comprehend and complete questionnaires in Swedish, and (ii) histopathologically and clinically confirmed CTCL diagnosis.

### Questionnaires regarding educational needs

The patients were asked to complete a questionnaire to capture their educational needs in regard to CTCL. The questionnaire was inspired by the educational needs assessment tool (ENAT), designed to help patients with rheumatoid arthritis identify their educational needs (13). ENAT has been validated and translated into several languages, and used in Swedish studies investigating educational needs among patients with rheumatic diseases (14). The questionnaire was adapted for the purpose of the present study and used several of the original questions (see **Supplementary 1**), translated from Swedish to English, with the original Swedish questionnaire attached. Responses to the question “In general, how much information do you want to receive about your lymphoma disease?” were given on a four-point Likert-type scale: nothing=0, some=1, a lot=2, everything=3. Five domains were used in the main body of the questionnaire, which covered information relating to disease process (6 items), treatment (4 items), feelings (2 items, regarding ways to manage stress and depression), self-management of itch, sleep, and rest (2 items), and support systems (3 items). Responses were given on a Likert-type scale, where the patients ranked the items as not important=0, a little important=1, very important=2, or extremely important=3. The domain scores ranged from 0 to a maximum of 18. A total score was calculated for each patient by adding up their responses in all five domains. This gave a total score from 0 to 51, with a higher score indicating a greater need for education. The five domains contained different numbers of items. Therefore, to facilitate comparisons between domains, each domain score was presented as a mean percentage of the maximum possible domain score.

### Variables

In addition to demographic characteristics gathered through the questionnaire, including sex, age, and education, the following clinical data were collected from the register: CTCL subtype, disease duration, clinical stage, activity as measured with the modified severity-weighted assessment tool (mSWAT) (15), and current treatment for CTCL. High educational level was defined as >12 years and low education as <12 years (completed primary school or high school).

### Statistical analysis

Baseline characteristics were expressed in proportions (%) of the total number of individuals observed and continuous data as medians with ranges. All percentages are presented as integrals; thus, the sums do not always add up to 100. The Mann-Whitney U-test was used to compare domain scores for variables categorized into two groups: sex, disease duration (<2 years vs. ≥2 years), education level, and clinical stage (IA vs. IB). The correlations between domains were determined with Spearman’s rank order correlation. Significance was defined as a p-value <0.05. All analyses were performed using SPSS, version 20 (IBM, Armonk, NY, USA).

### Ethics

The study was approved by the Swedish Ethics Review Authority (2019-03467). Verbal and written consent was obtained from the patients before inclusion.

## Results

### Baseline patient characteristics

All CTCL patients in the local cutaneous lymphoma register were included in the study (n=70), females represented 36% (**Table 1**). The median age was 68 years (range 27–90). The mean disease duration was 9.1 years (standard deviation: 11.6), with a range of 0–52 years. More than 70% had been diagnosed with CTCL for more than 2 years before inclusion in this study. The majority were diagnosed with MF (n=55), followed by lymphomatoid papulosis (n=10), and other CTCL (n=5), including primary cutaneous anaplastic large cell lymphoma (n=2), primary cutaneous peripheral T-cell lymphoma unspecified (n=2), and Sézary syndrome (n=1). Among the patients with MF, most had early-stage disease (stages IA–IIA: n=51 (93%)) with low mSWAT scores (median 4, range 0–70). Higher education was reported by 63% of the patients.

**Table 1:** Patient demographics and grouping variables, n=70.

Characteristics	Value
Age, mean (SD), range, median (IQR), years	63.0 (15.1), 27–90, 68 (23)
Gender, n (%)	
Females	25 (36)
Males Diagnosis, n (%)	45 (64)
Mycosis fungoides	55 (79)
Lymphomatoid papulosis	10 (14)
Other^a^	5 (7)
Disease duration^b^, mean (SD), median (range), years	9.1 (11.6), 4.5 (0–52)
<2 years, n (%)	20 (29)
≥2 years, n (%)	50 (71)
Current clinical stage for patients with MF^c^, n (%)	
IA	36 (66)
IB	15 (27)
IIA	1 (2)
IIB	2 (4)
IIIB	1 (2)
mSWAT, mean (SD), median (range)	12.8 (17.2), 4 (0–70)
Treatment, n (%)	
None +/- emollients	19 (27)
Skin-directed therapy	42 (60)
PUVA or UVB +/- topical steroids	6 (14)
Topical steroids	36 (86)
Systemic therapy^d^	9 (13)
Education, n (%)	
Primary school or high school	26 (37)
Higher education	44 (63)

^a^Primary cutaneous anaplastic large cell lymphoma (n=2), Sézary syndrome (n=1), primary cutaneous peripheral T-cell lymphoma unspecified (n=2). ^b^Years since diagnosis. ^c^According to the tumor-node-metastasis-blood clinical stage classification. ^d^Systemic therapies: acitretin n=3, alitretinoin n=2, prednisolone n=1, methotrexate n=2, alemtuzumab n=1.

IQR, interquartile range; MF, mycosis fungoides; mSWAT, modified severity-weighted assessment tool; PUVA, psoralen and ultraviolet A-type light; SD, standard deviation; UVB, ultraviolet B-type light.

### Overall self-reported educational needs

When asked “In general, how much information do you need?”, females indicated greater interest in information than males (2.6 vs. 2.1, p=0.006), and patients with higher education indicated greater interest than patients with lower education (2.5 vs. 2.0, *p=*0.025) (**Table 2**). The answers did not differ significantly depending on disease duration (<2 years vs. ≥ 2 years). The same patterns were observed in a sub-analysis of patients with MF (**Table 3**).

**Table 2:** Overall informational needs and educational needs assessment tool domains among 70 patients with cutaneous T-cell lymphoma*.

	All (n=70)	Females (n=25)	Males (n=45)	p-value	Disease duration <2 years (n=20)	Disease duration ≥2 years (n=50)	p-value	Primary school or high school (n=26)	Higher education (n=44)	p-value
Overall informational needs^a^ 0–3	2.3 (0.83) (76%)	2.6 (0.6) (88%)	2.1 (0.9) (69%)	**0.006**	2.1 (0.9) (70%)	2.3 (0.8) (78%)	0.316	2.0 (0.9) (65%)	2.5 (0.7) (82%)	**0.025**
Domains										
Disease process 0–18	13.5 (4.4) (75%)	15.0 (3.8) (83%)	12.7 (4.5) (71%)	**0.030**	14.6 (4.1) (81%)	13.1 (4.5) (72%)	0.099	12.9 (4.7) (72%)	13.9 (4.2) (77%)	0.481
Treatments^b^ 0–12	9.6 (3.1) (80%)	10.6 (2.4) (88%)	9.0 (3.3) (75%)	**0.024**	10.7 (1.9) (89%)	9.1 (3.4) (76%)	0.117	9.7 (3.0) (81%)	9.5 (3.2) (79%)	0.948
Feelings 0–6	2.6 (2.2) (44%)	3.1 (2.2) (52%)	2.3 (2.2) (39%)	0.150	2.9 (2.4) (48%)	2.5 (2.2) (42%)	0.511	3.3 (2.1) (54%)	2.2 (2.2) (37%)	**0.049**
Self-management 0–6	3.4 (2.0) (57%)	3.9 (1.6) (65%)	3.2 (2.1) (52%)	0.178	3.8 (2.2) (63%)	3.3 (1.9) (54%)	0.232	3.8 (2.1) (64%)	3.2 (1.8) (52%)	0.100
Support systems 0–9	3.7 (2.2) (41%)	4.3 (1.9) (48%)	3.4 (2.4) (37%)	**0.039**	4.7 (2.1) (52%)	3.3 (2.2) (36%)	**0.017**	4.3 (2.4) (48%)	3.3 (2.1) (37%)	0.085
Total score** 0–51	33.0 (11.1) (65%)	36.8 (8.9) (72%)	30.8 (11.7) (61%)	**0.032**	37.6 (8.5) (74%)	31.2 (11.6) (61%)	0.051	34.7 (11.6) (68%)	32.0 (10.8) (63%)	0.303

*Cutaneous T-cell lymphomas include mycosis fungoides (n=55), lymphomatoid papulosis (n=10), primary cutaneous anaplastic large cell lymphoma (n=2), Sézary syndrome (n=1), and primary cutaneous peripheral T-cell lymphoma unspecified (n=2) ^a^In general, how much information do you want to receive about your lymphoma disease? (1 question ^b^Treatments, missing n=1 (in 1/4 questions). **Including all domains, missing n=1. Presented as mean score (standard deviation) and mean percentage of maximum score. Statistically significant values are marked in bold.

**Table 3:** Overall informational needs and educational needs assessment tool domains among 55 patients with mycosis fungoides.

	All** (n=55)	Females (n=20)	Males (n=35)	p-value	Disease duration <2 years (n=14)	Disease duration ≥2 years (n=41)	p-value	Primary school or high school (n=20)	Higher education (n=35)	p-value
Overall informational needs^a^ 0–3	2.4 (0.76)	2.9 (0.4) (95%)	2.2 (0.8) (72%)	**0.001**	2.2 (0.9) (73%)	2.5 (0.7) (83%)	0.315	2.1 (0.9) (70%)	2.6 (0.6) (87%)	**0.041**
Domains										
Disease process 0–18	13.8 (4.5) (77%)	15.7 (3.4) (87%)	12.7 (4.7) (70%)	**0.008**	15.1 (3.8) (84%)	13.3 (4.6) (74%)	0.105	13.1 (5.0) (73%)	14.2 (4.2) (79%)	0.508
Treatments 0–12	9.8 (3.2) (81%)	11.0 (2.1) (92%)	9.1 (3.5) (75%)	**0.015**	10.9 (1.6) 91%	9.4 (3.5) (78%)	0.208	9.6 (3.1) (80%)	9.9 (3.3) (82%)	0.507
Feelings 0–6	2.7 (2.3) (45%)	3.2 (2.3) (53%)	2.4 (2.2) (40%)	0.239	3.3 (2.4) (55%)	2.5 (2.2) (42%)	0.261	3.4 (2.1) (56%)	2.3 (2.3) (39%)	0.092
Self-management 0–6	3.7 (1.9) (61%)	4.2 (1.4) (70%)	3.4 (2.1) (57%)	0.232	4.6 (1.7) (76%)	3.4 (1.9) (57%)	**0.045**	4.2 (2.1) (69%)	3.4 (1.8) (57%)	0.127
Support systems 0–9	3.7 (2.3) (41%)	4.4 (2.0) (49%)	3.3 (2.4) (37%)	0.054	5.1 (2.2) (56%)	3.2 (2.2) (36%)	**0.009**	4.5 (2.6) (49%)	3.3 (2.1) (37%)	0.080
Total score* 0–51	33.7 (11.6) (66%)	38.5 (8.2) (75%)	30.9 (12.4) (61%)	**0.021**	39.0 (8.7) (77%)	31.8 (11.9) (62%)	0.052	34.7 (12.5) (68%)	33.1 (11.1) (65%)	0.557

^a^In general, how much information do you want to receive about your lymphoma disease? (1 question). *Including all domains. Presented as mean score (standard deviation) and mean percentage of maximum score. Statistically significant values are marked in bold.

### Domains in the overall CTCL cohort

Exploration of each domain regarding the percentage of maximum domain score revealed different degrees of educational needs. The domains concerning treatment (80%) and disease process (75%) revealed the greatest need for education in CTCL patients, whereas self-management (57%), feelings (44%), and support systems (41%) revealed the smallest needs for education (**Table 2**). The total score was higher among females than males, but not significantly different when explored in relationship to disease duration and education (**Table 2**). Females reported greater educational needs in the domains disease process, treatment, and support systems than males. Patients with a disease duration <2 years reported greater educational needs for the domain support system compared with patients with longer disease duration. Patients with lower education reported greater educational needs about feelings compared with patients with higher education. There was a positive correlation between all domains (i.e., disease process, treatment, feelings, self-management, and support systems), with the strongest correlation observed between disease process and treatment (r=0.761, p=0.001). A weak correlation was observed between disease process and feelings (r=0.295, p=0.013). No correlation was found between age and total score. One patient with lymphomatoid papulosis did not complete data in the treatment domain (missing=1/4 questions) but responded in all other domains. Data from this patient were therefore excluded in the total scores and the treatment domain.

### Domains in the MF cohort

In a sub-analysis among patients with MF (n=55), similar findings as those described for the overall CTCL cohort were made (**Table 3**). A greater informational need regarding self-management was observed in patients with <2 years disease than in patients with longer disease duration. There was a trend towards greater educational needs regarding feelings among MF patients with lower educational levels, but the difference did not reach significance (p=0.092). Among patients with early-stage MF (n=51), there was no significant difference in total score between patients in stage IA (n=36) and IB (n=15) (34.1 vs. 31.6, p=0.748).

## Discussion

To the best of our knowledge, the data on educational needs among patients with CTCL are limited and have not previously been investigated in detail. This cross-sectional study showed that patients with CTCL have considerable educational needs. The mean total score was 33.0, corresponding to 65% of the maximum score, with the highest scores found for the disease process and treatment domains. Females seemed to have greater educational needs concerning disease process, treatment, and support systems than males. Patients with higher education levels to a higher degree answered that they wanted to know “everything” about the disease. In a sub-analysis among patients with MF, similar patterns were found.

In line with our results, a systematic review exploring the unmet supportive care needs of patients with hematological malignancies (lymphoma, leukemia, and lymphoma; CTCL were not included) found that the most frequently reported unmet supportive care needs were informational needs, followed by psychological/emotional needs and functional needs. Patients were most concerned about obtaining information about their future health (16). In the present study, we found a strong correlation between educational needs regarding disease process and treatment, indicating unmet educational needs in this group. CTCL patients with lower educational levels expressed a need for more education about managing stress and depression, compared with patients with higher education levels. This finding is clinically important and underlines the need of individual management in daily practice. CTCL can have a detrimental impact on QoL with significant impairment of functional, emotional, and physical aspects (2, 3). Future studies are needed to determine if CTCL patients with lower education levels have an increased vulnerability to stress and depressive symptoms and are particularly prone to suffer from impaired QoL.

Our findings indicated that patients’ educational needs regarding support systems and management decreased over time among patients with MF. We speculate that this is, in part, a result of deepened understanding of the disease over time, likely in part related to information given at clinical visits. To better understand these aspects, future studies should include a longitudinal analysis of the educational needs in CTCL over time. Such dynamic evaluation could reveal the effect of treatment responses and disease progression on educational needs in CTCL patients. The majority of patients had high education levels and were receptive to information about the disease, as shown by the total scores.

The study had several limitations. There are differences in the clinical manifestations, treatment, and prognosis of the different subtypes of CTCL, and some confounders could not be adjusted for due to the small number of participants in some subtypes. More studies are needed to confirm the results in advanced CTCL stages. In addition, we did not have information about whether the patients had sought information about CTCL themselves. Furthermore, we designed the questionnaire for the purpose of the present study and it was not validated. It likely did not fully capture the educational needs of CTCL patients, but hopefully captured the overall trends. In support of this, the highest total scores were found for the disease process and treatment domains, which is congruent with the results for a previous item used in a validation study of QoL in CTCL (9). The strengths of the study included the novel insights into educational needs in this understudied patient group and the fairly large number of patients with MF, enabling a sub-analysis of patients with this CTCL subtype.

## Conclusions

CTCL patients in the cohort, particularly females, expressed a need for education, especially regarding disease process and treatment. A deeper understanding of educational needs would enable healthcare providers to give personalized information about the disease as a main supportive component. Further studies are needed to formulate and validate an assessment tool for capturing educational needs among patients with CTCL, as well as to explore longitudinal data.

## Data availability statement

The raw data supporting the conclusions of this article will be made available by the authors, upon reasonable request.

## Ethics statement

The studies involving humans were approved by The Ethics Review Authority Drottninggatan 7 S-753 10 Uppsala Sweden. The studies were conducted in accordance with the local legislation and institutional requirements. The participants provided their written informed consent to participate in this study.

## Author contributions

LI: Data curation, Formal analysis, Investigation, Methodology, Visualization, Writing – original draft. AW: Data curation, Formal analysis, Investigation, Writing – review & editing. PJ: Data curation, Investigation, Writing – review & editing. HB: Conceptualization, Data curation, Funding acquisition, Investigation, Project administration, Resources, Supervision, Writing – original draft.
