# Corrigendum: Exploring the educational needs of patients with cutaneous lymphoma using an educational needs assessment tool

**DOI:** 10.3389/fonc.2024.1504102

**Published:** 2024-12-11

**Authors:** Lina U. Ivert, Anna H. Winther, Pontus Jonsson, Hanna Brauner

**Affiliations:** ^1^ Dermatology and Venereology Unit, Department of Medicine Solna, Karolinska Institutet, Stockholm, Sweden; ^2^ Department of Dermatology, Karolinska University Hospital, Stockholm, Sweden

**Keywords:** mycosis fungoides (MF), cutaneous T-cell lymphoma (CTCL), educational needs, patient education, self-reported questionnaire

In the published article, there was an error in **Table 1** as published. The caption “Disease duration^b^” was incorrectly written as “Disease durationb”. The corrected **Table 1** and its caption “Disease duration^b^” appear below.

**Table 1:** Patient demographics and grouping variables, n=70.

Characteristics	Value
Age, mean (SD), range, median (IQR), years	63.0 (15.1), 27–90, 68 (23)
Gender, n (%)	
Females	25 (36)
Males Diagnosis, n (%)	45 (64)
Mycosis fungoides	55 (79)
Lymphomatoid papulosis	10 (14)
Other^a^	5 (7)
Disease duration^b^, mean (SD), median (range), years	9.1 (11.6), 4.5 (0–52)
<2 years, n (%)	20 (29)
≥2 years, n (%)	50 (71)
Current clinical stage for patients with MF^c^, n (%)	
IA	36 (66)
IB	15 (27)
IIA	1 (2)
IIB	2 (4)
IIIB	1 (2)
mSWAT, mean (SD), median (range)	12.8 (17.2), 4 (0–70)
Treatment, n (%)	
None +/- emollients	19 (27)
Skin-directed therapy	42 (60)
PUVA or UVB +/- topical steroids	6 (14)
Topical steroids	36 (86)
Systemic therapy^d^	9 (13)
Education, n (%)	
Primary school or high school	26 (37)
Higher education	44 (63)

^a^Primary cutaneous anaplastic large cell lymphoma (n=2), Sézary syndrome (n=1), primary cutaneous peripheral T-cell lymphoma unspecified (n=2). ^b^Years since diagnosis. ^c^According to the tumor-node-metastasis-blood clinical stage classification. ^d^Systemic therapies: acitretin n=3, alitretinoin n=2, prednisolone n=1, methotrexate n=2, alemtuzumab n=1.

IQR, interquartile range; MF, mycosis fungoides; mSWAT, modified severity-weighted assessment tool; PUVA, psoralen and ultraviolet A-type light; SD, standard deviation; UVB, ultraviolet B-type light.

“Disease duration^b^.”

In the published article, there was an error in **Tables 2** and **3** as published. “Presented as mean score (standard deviation) and (mean percentage of maximum score)” should be changed to “Presented as mean score (standard deviation) and mean percentage of maximum score”. The corrected **Tables 2** and **3** and their captions appear below.

**Table 2:** Overall informational needs and educational needs assessment tool domains among 70 patients with cutaneous T-cell lymphoma*.

	All (n=70)	Females (n=25)	Males (n=45)	p-value	Disease duration <2 years (n=20)	Disease duration ≥2 years (n=50)	p-value	Primary school or high school (n=26)	Higher education (n=44)	p-value
Overall informational needs^a^0–3	2.3 (0.83) (76%)	2.6 (0.6) (88%)	2.1 (0.9) (69%)	**0.006**	2.1 (0.9) (70%)	2.3 (0.8) (78%)	0.316	2.0 (0.9) (65%)	2.5 (0.7) (82%)	**0.025**
Domains										
Disease process 0–18	13.5 (4.4) (75%)	15.0 (3.8) (83%)	12.7 (4.5) (71%)	**0.030**	14.6 (4.1) (81%)	13.1 (4.5) (72%)	0.099	12.9 (4.7) (72%)	13.9 (4.2) (77%)	0.481
Treatments^b^ 0–12	9.6 (3.1) (80%)	10.6 (2.4) (88%)	9.0 (3.3) (75%)	**0.024**	10.7 (1.9) (89%)	9.1 (3.4) (76%)	0.117	9.7 (3.0) (81%)	9.5 (3.2) (79%)	0.948
Feelings 0–6	2.6 (2.2) (44%)	3.1 (2.2) (52%)	2.3 (2.2) (39%)	0.150	2.9 (2.4) (48%)	2.5 (2.2) (42%)	0.511	3.3 (2.1) (54%)	2.2 (2.2) (37%)	**0.049**
Self-management 0–6	3.4 (2.0) (57%)	3.9 (1.6) (65%)	3.2 (2.1) (52%)	0.178	3.8 (2.2) (63%)	3.3 (1.9) (54%)	0.232	3.8 (2.1) (64%)	3.2 (1.8) (52%)	0.100
Support systems 0–9	3.7 (2.2) (41%)	4.3 (1.9) (48%)	3.4 (2.4) (37%)	**0.039**	4.7 (2.1) (52%)	3.3 (2.2) (36%)	**0.017**	4.3 (2.4) (48%)	3.3 (2.1) (37%)	0.085
Total score** 0–51	33.0 (11.1) (65%)	36.8 (8.9) (72%)	30.8 (11.7) (61%)	**0.032**	37.6 (8.5) (74%)	31.2 (11.6) (61%)	0.051	34.7 (11.6) (68%)	32.0 (10.8) (63%)	0.303

*Cutaneous T-cell lymphomas include mycosis fungoides (n=55), lymphomatoid papulosis (n=10), primary cutaneous anaplastic large cell lymphoma (n=2), Sézary syndrome (n=1), and primary cutaneous peripheral T-cell lymphoma unspecified (n=2) ^a^In general, how much information do you want to receive about your lymphoma disease? (1 question ^b^Treatments, missing n=1 (in 1/4 questions). **Including all domains, missing n=1. Presented as mean score (standard deviation) and mean percentage of maximum score. Statistically significant values are marked in bold.

**Table 3:** Overall informational needs and educational needs assessment tool domains among 55 patients with mycosis fungoides.

	All** (n=55)	Females (n=20)	Males (n=35)	p-value	Disease duration <2 years (n=14)	Disease duration ≥2 years (n=41)	p-value	Primary school or high school (n=20)	Higher education (n=35)	p-value
Overall informational needs^a^ 0–3	2.4 (0.76)	2.9 (0.4) (95%)	2.2 (0.8) (72%)	**0.001**	2.2 (0.9) (73%)	2.5 (0.7) (83%)	0.315	2.1 (0.9) (70%)	2.6 (0.6) (87%)	**0.041**
Domains										
Disease process 0–18	13.8 (4.5) (77%)	15.7 (3.4) (87%)	12.7 (4.7) (70%)	**0.008**	15.1 (3.8) (84%)	13.3 (4.6) (74%)	0.105	13.1 (5.0) (73%)	14.2 (4.2) (79%)	0.508
Treatments 0–12	9.8 (3.2) (81%)	11.0 (2.1) (92%)	9.1 (3.5) (75%)	**0.015**	10.9 (1.6) 91%	9.4 (3.5) (78%)	0.208	9.6 (3.1) (80%)	9.9 (3.3) (82%)	0.507
Feelings 0–6	2.7 (2.3) (45%)	3.2 (2.3) (53%)	2.4 (2.2) (40%)	0.239	3.3 (2.4) (55%)	2.5 (2.2) (42%)	0.261	3.4 (2.1) (56%)	2.3 (2.3) (39%)	0.092
Self-management 0–6	3.7 (1.9) (61%)	4.2 (1.4) (70%)	3.4 (2.1) (57%)	0.232	4.6 (1.7) (76%)	3.4 (1.9) (57%)	**0.045**	4.2 (2.1) (69%)	3.4 (1.8) (57%)	0.127
Support systems 0–9	3.7 (2.3) (41%)	4.4 (2.0) (49%)	3.3 (2.4) (37%)	0.054	5.1 (2.2) (56%)	3.2 (2.2) (36%)	**0.009**	4.5 (2.6) (49%)	3.3 (2.1) (37%)	0.080
Total score* 0–51	33.7 (11.6) (66%)	38.5 (8.2) (75%)	30.9 (12.4) (61%)	**0.021**	39.0 (8.7) (77%)	31.8 (11.9) (62%)	0.052	34.7 (12.5) (68%)	33.1 (11.1) (65%)	0.557

^a^In general, how much information do you want to receive about your lymphoma disease? (1 question). *Including all domains. Presented as mean score (standard deviation) and mean percentage of maximum score. Statistically significant values are marked in bold.

“Presented as mean score (standard deviation) and mean percentage of maximum score.”

A correction has been made to the **Abstract**, *Methods*. This sentence previously stated:

“The domain scores ranged from 0 to 18 and the total score from 0 to 51, with a higher score indicating a greater need for education.”

The corrected sentence appears below:

“The domain scores ranged from 0 to 18 (total score from 0 to 51). Each domain score was presented as a mean percentage of the maximum possible domain score.”

A correction has been made to the **Abstract**, *Conclusion*. This sentence previously stated:

“We found that 65% of the CTCL patients in the cohort, particularly females, expressed a need for education, especially regarding disease process and treatment.”

The corrected sentence appears below:

“CTCL patients in the cohort, particularly females, expressed a need for education, especially regarding disease process and treatment.”

A correction has been made to the Main text*, Questionnaires regarding educational needs.* This sentence previously stated:

“Therefore, to facilitate comparisons between domains, the total score was divided by the maximum possible score and presented as a mean percentage.”

The corrected sentence appears below:

“Therefore, to facilitate comparisons between domains, each domain score was presented as a mean percentage of the maximum possible domain score.”

A correction has been made to the Main text*, Questionnaires regarding educational needs (last sentence).* This sentence previously stated:

“The questionnaire was adapted for the purpose of the present study and used several of the original questions (see Supplementary 1), translated from Swedish to English, with the original Swedish questionnaire attached).”

The corrected sentence appears below:

“The questionnaire was adapted for the purpose of the present study and used several of the original questions (see Supplementary 1), translated from Swedish to English, with the original Swedish questionnaire attached.”

A correction has been made to the Main text*, Result, overall self-reported educational needs.*

This sentence previously stated:

“The answers did not differ significantly depending on disease duration (<2 years vs. = 2 years).”

The corrected sentence appears below:

“The answers did not differ significantly depending on disease duration (<2 years vs. ≥ 2 years).”

A correction has been made to the Main text, **Conclusion.** This sentence previously stated:

“We found that 65% of the CTCL patients in the cohort, particularly females, expressed a need for education, especially regarding disease process and treatment.”

The corrected sentence appears below:

“CTCL patients in the cohort, particularly females, expressed a need for education, especially regarding disease process and treatment.”

The authors apologize for these errors and state that this does not change the scientific conclusions of the article in any way. The original article has been updated.

