# Supplementary material for: Exploring the educational needs of patients with cutaneous lymphoma using an educational needs assessment tool
Source: Front Oncol. 2024 Aug 7;14:1433821. doi: 10.3389/fonc.2024.1433821 (PMC11335495; doi:10.3389/fonc.2024.1433821)
Supplement: Supplementary file 1 [file DataSheet1.pdf]

Questionnaire regarding informational needs for cutaneous lymphoma

Please respond to the following questions

I am: Man ( )

Woman ( )

How old are you? ………………………

How many years have you had cutaneous lymphoma? ………………………

How long an education do you have (including elementary school)? ………………………

Do you, right NOW, need information about something that could help you with your cutaneous lymphoma disease?

Yes ( )

No ( )

If yes, what information do you need?

In general, how much information do you want about your cutaneous lymphoma disease?

I don't want to know anything ( )

I want to know some ( )

I want to know a lot ( )

I want to know everything ( )

How much do you need to know NOW about the following areas?

For each area, tick the box you think is most appropriate.

This part is about your cutaneous lymphoma disease.

How important is it for you to know more about the following:	Not important	A little important	Very important	Extremely important
What could have caused my cutaneous lymphoma	( )	( )	( )	( )
Which type of cutaneous lymphoma I have	( )	( )	( )	( )
How the disease can be treated	( )	( )	( )	( )
How the disease can affect my children or relatives	( )	( )	( )	( )
How the disease affects me	( )	( )	( )	( )
What can happen in the future	( )	( )	( )	( )

This part is about treatment you can get from healthcare personnel.

How important it is for you to know more about the following:	Not important	A little important	Very important	Extremely important
Why I'm getting treatment	( )	( )	( )	( )
How the treatment should be performed	( )	( )	( )	( )
What side effects the treatment can cause	( )	( )	( )	( )
Why I am giving blood samples	( )	( )	( )	( )

This part is about ways to manage itch, about your feelings and about activities.

How important it is for you to know more about the following:	Not important	A little important	Very important	Extremely important
Ways to manage itch	( )	( )	( )	( )
Ways to get enough sleep and rest	( )	( )	( )	( )
Ways to manage stress	( )	( )	( )	( )
Ways to manage depression	( )	( )	( )	( )

This part is about support from other people.

How important it is for you to know more about the following:	Not important	A little important	Very important	Extremely important
Organisations that I can contact regarding my disease	( )	( )	( )	( )
Who I can ask for financial help	( )	( )	( )	( )
How I can get the most out of my visits to my physician or nurse	( )	( )	( )	( )

Thank you for taking the time to fill out this questionnaire.

Frågeformulär om behov av information vid hudlymfom

Var snäll och besvara följande frågor

**Jag är:**	**Man**	**( )**
	**Kvinna**	**( )**

Hur gammal är du? ....

Hur många år har du haft hudlymfom? ....

Hur lång utbildning har du (inklusive grundskola)? ....

Skulle du just <u>NU</u> behöva information om något som kan hjälpa dig med din hudlymfom-sjukdom?

Ja ( )

Nej ( )

Om ja, vad?

I allmänhet, hur mycket information vill du få om din hudlymfom-sjukdom?

**Jag vill inte veta någonting**	**( )**
**Jag vill veta en del**	**( )**
**Jag vill veta mycket**	**( )**
**Jag vill veta allt**	**( )**

Hur mycket behöver du veta NU om följande områden?
Pricka för varje område i den ruta som du tycker passar bäst.

Den här delen handlar din hudlymfom-sjukdom.

**Hur viktigt är det för dig att veta mer om följande:**	**Inte alls viktigt**	**Lite viktigt**	**Mycket viktigt**	**Väldigt viktigt**
Vad som kan ha orsakat min hudlymfom-sjukdom	( )	( )	( )	( )
Vilken sorts hudlymfom jag har	( )	( )	( )	( )
Hur sjukdomen kan behandlas	( )	( )	( )	( )
Hur sjukdomen kan påverka mina barn eller anhöriga	( )	( )	( )	( )
Hur sjukdomen påverkar mig	( )	( )	( )	( )
Vad som kan hända i framtiden	( )	( )	( )	( )

Den här delen handlar om behandlingar du kan få av vårdpersonal.

**Hur viktigt är det för dig att veta mer om följande:**	**Inte alls viktigt**	**Lite viktigt**	**Mycket viktigt**	**Väldigt viktigt**
Varför jag får behandling	( )	( )	( )	( )
Hur behandlingen ska utföras	( )	( )	( )	( )
Vilka biverkningar behandlingen kan ge	( )	( )	( )	( )
Varför jag tar blodprov	( )	( )	( )	( )

Den här delen handlar om att hantera klåda, om dina känslor och om aktivitet.

**Hur viktigt är det för dig att veta mer om följande:**	**Inte alls viktigt**	**Lite viktigt**	**Mycket viktigt**	**Väldigt viktigt**
Sätt att hantera klåda	( )	( )	( )	( )
Sätt att få tillräckligt med sömn och vila	( )	( )	( )	( )
Sätt att hantera stress	( )	( )	( )	( )
Sätt att hantera nedstämdhet	( )	( )	( )	( )

Den här delen handlar om stöd från andra människor.

**Hur viktigt är det för dig att veta mer om följande:**	**Inte alls viktigt**	**Lite viktigt**	**Mycket viktigt**	**Väldigt viktigt**
Organisationer som jag kan kontakta angående min sjukdom	( )	( )	( )	( )
Vem jag kan fråga om ekonomisk hjälp	( )	( )	( )	( )
Hur jag kan få ut så mycket som möjligt av mina besök hos läkare eller sköterska	( )	( )	( )	( )

Tack för att du tagit dig tid att fylla i detta formulär.
